# The Graded Change in Connectivity across the Ventromedial Prefrontal Cortex Reveals Distinct Subregions

**DOI:** 10.1093/cercor/bhz079

**Published:** 2019-04-26

**Authors:** Rebecca L Jackson, Claude J Bajada, Matthew A Lambon Ralph, Lauren L Cloutman

**Affiliations:** 1 Medical Research Council Cognition & Brain Sciences Unit, University of Cambridge, Cambridge, UK; 2 Faculty of Medicine and Surgery, University of Malta, Msida, MSD, Malta; 3 Neuroscience and Aphasia Research Unit (NARU), Division of Neuroscience & Experimental Psychology (Zochonis Building), University of Manchester, Manchester, UK

**Keywords:** default mode network, functional connectivity, orbitofrontal cortex, parcellation, tractography

## Abstract

The functional heterogeneity of the ventromedial prefrontal cortex (vmPFC) suggests it may include distinct functional subregions. To date these have not been well elucidated. Regions with differentiable connectivity (and as a result likely dissociable functions) may be identified using emergent data-driven approaches. However, prior parcellations of the vmPFC have only considered hard splits between distinct regions, although both hard and graded connectivity changes may exist. Here we determine the full pattern of change in structural and functional connectivity across the vmPFC for the first time and extract core distinct regions. Both structural and functional connectivity varied along a dorsomedial to ventrolateral axis from relatively dorsal medial wall regions to relatively lateral basal orbitofrontal cortex. The pattern of connectivity shifted from default mode network to sensorimotor and multimodal semantic connections. This finding extends the classical distinction between primate medial and orbital regions by demonstrating a similar gradient in humans for the first time. Additionally, core distinct regions in the medial wall and orbitofrontal cortex were identified that may show greater correspondence to functional differences than prior hard parcellations. The possible functional roles of the orbitofrontal cortex and medial wall are discussed.

## Introduction

The ventromedial prefrontal cortex (vmPFC) is a complex region postulated to relate to multiple different functions, including semantic cognition, affect, reward, decision making and social cognition (Mah et al. 2005; Binder et al. 2009; Rushworth et al. 2011; Winecoff et al. 2013). It is also considered a core region of the default mode network (DMN); a set of functionally-connected regions that deactivate for various tasks (Buckner et al. 2008) with postulated involvement in episodic memory, mind-wandering, internally-directed attention, social cognition and mental simulation (Mason et al. 2007; Buckner et al. 2008; Mars et al. 2012). The functional heterogeneity of the vmPFC indicates that it may consist of differentiable functional subregions, yet its organization remains unclear. This may be as most research has been conducted within one domain only, resulting in research fractionation. An additional obstacle to distinguishing functional subregions is the signal loss and distortion present within the vmPFC in fMRI due to the proximity of air-filled cavities (Embleton et al. 2010; Halai et al. 2014). Although this is a critical problem when assessing the current literature, contemporary fMRI studies can resolve this issue by taking advantage of recently established multi-echo acquisition methods capable of maintaining signal in the problematic areas including the vmPFC, without loss of signal elsewhere (Poser et al. 2006; Poser et al. 2009; Halai et al. 2014).

**Figure 1:**
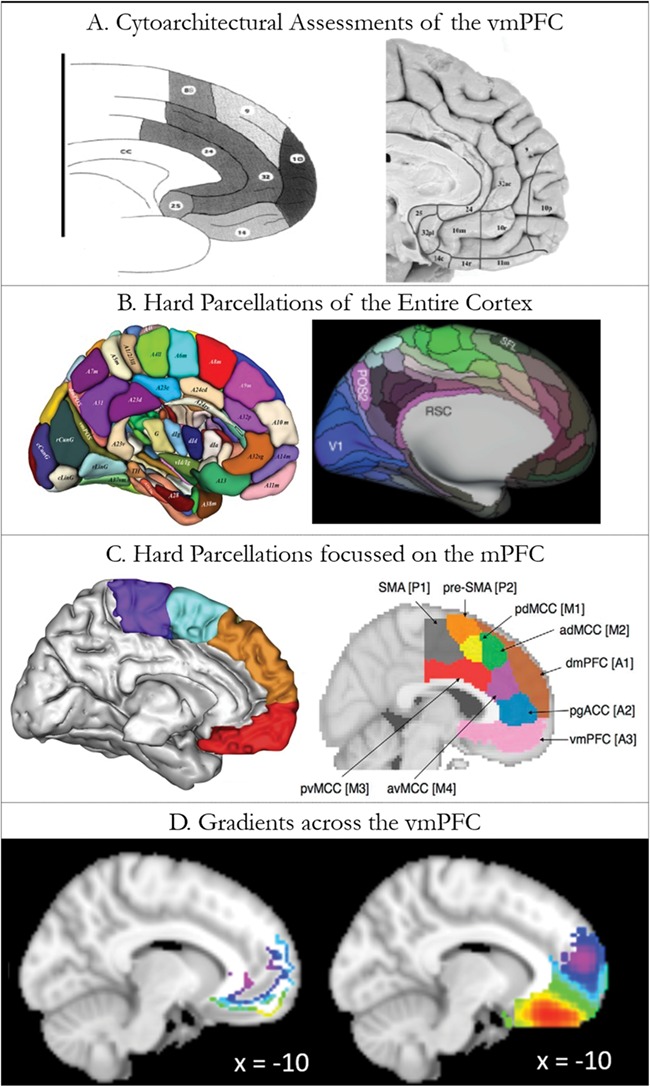
A diagrammatic review of some of the key literature on divisions within the vmPFC. A. The cytoarchitectural divisions within the vmPFC. Öngür et al. (2003) displayed the divisions discovered in Petrides & Pandya (1994; left) and their own updated divisions (on the right; Öngür et al. (2003). B. Recent hard parcellations of the entire cortex. The left panel shows Fan et al.’s (2016) division based on multimodal imaging techniques and the right panel displays Glasser et al.’s (2016) multimodal parcellation. C. Hard parcellations focussed on the mPFC. The left panel is Thiebaut de Schotten et al.’s (2016) tractographic parcellation and the right shows De la Vega et al.’s (2016) 9-cluster solution based on functional co-activation. The labels are from the original diagram. D. The current findings are provided for comparison. The left shows the structural connectivity gradient and the right shows the functional connectivity gradient. Figures are reproduced with permission from the copyright holders.

The function of a cortical area is critically dependent on its connectivity, which determines the nature and flow of information to and from an area (Brodmann 1909; Plaut 2002; Cloutman et al. 2012; Chen et al. 2017; Behrens et al. 2005). As such, many recent research efforts aimed at understanding the complex organization of functionally diverse brain regions have focused on the delineation of their structural and/or functional connections. Emergent, data-driven approaches may be used to group voxels with similar connectivity patterns in a bottom-up fashion without a dependence on seed regions. Both graded and discrete changes in structural and functional connectivity exist within the brain, and intermediary regions that show a more gradual transition in connective profile, may exist between regions with more distinct connectivity patterns (Brodmann 1909; Cerliani et al. 2012; Margulies et al. 2016; Bajada et al. 2017b; Braga et al. 2017; Haak et al. 2018; Jackson et al. 2018). A small number of studies have used hard parcellation techniques to distinguish regions of the vmPFC based on its structural or functional connectivity (see Figure 1; de la Vega et al. 2016; Thiebaut de Schotten et al. 2016), as well as hard parcellations of the entire cortex (see Figure 1; Fan et al. 2016; Glasser et al. 2016). However, traditional hard parcellation techniques presuppose sharp divisions and therefore force each voxel into a cluster. As such, voxels within intermediate regions will be grouped with a specific cluster despite their lack of distinct connectivity profiles. Consequently, core regions with distinct connectivity may not be well delineated and the connectivity of the overall clusters will be less distinct (Haueis 2012; Bajada et al. 2017b). An alternative approach is to examine in detail the change in functional or structural connectivity pattern across an entire region, thus visualizing all distinctions whether sharp or graded (Johansen-Berg et al. 2004; Cerliani et al. 2012; Margulies et al. 2016; Bajada et al. 2017b; Jackson et al. 2018). Spectral reordering approaches have previously been used to demonstrate both graded (Bajada et al. 2017b; Haak et al. 2018) and hard parcellations (Johansen-Berg et al. 2004) within the brain and do not presuppose the nature of the connectivity change. Core regions with distinct connectivity profiles can then be extracted and their connectivity determined. Spectral-reordering of tractography results has been shown to have high cross-validity with cytoarchitectural assessment (Klein et al. 2007; see Figure 1 for key cytoarchitectural divisions of the vmPFC). Accordingly, this approach was applied in this study separately to tractography data and dual-echo resting-state fMRI data (as per Jackson et al. 2018) to inform on the function of subregions and enable a direct comparison of the vmPFC regions structural and functional connective organization.

## Materials and Methods

### Participants

The structural connectivity analyses were performed using diffusion-weighted and structural (T1- and T2-weighted) MR images acquired from 24 healthy participants (11 female, aged 19–47 years, average 25.9 years) previously reported in Cloutman et al. (2012) and Binney et al. (2012). Functional connectivity analyses were performed on an independent resting-state dataset of 78 participants (57 female, age range 18–42, average age 24.71 years, SD 5.49 years), reported previously (Jackson et al. 2016; Jung et al. 2016; Jackson et al. 2018). In both cases participants were right-handed [as determined by the Edinburgh Handedness Inventory (Oldfield 1971)] and gave informed written consent. The study was approved by the local ethics board.

### Region of Interest

The two parcellation analyses used the same base region of interest (ROI) of the vmPFC although the structural analysis focused on the boundary between the gray and white matter, whereas the functional analyses included the entire region. The vmPFC is not a precise anatomical term and the extent of the area referred to as the vmPFC is variable. The dorsal edge may be considered to be horizontal at the approximate level of the genu or extend diagonally to cover the entire frontal pole, ending in line with Vogt’s (2004) cytoarchitectural division between subgenual and dorsal anterior cingulate (Etkin et al. 2011; Roy et al. 2012). Areas of the anterior cingulate cortex are often considered vmPFC and functional results may transcend the cingulate sulcus (e.g. Etkin et al. 2011; Desmet et al. 2015). Therefore, we chose to define the vmPFC inclusively in order to allow assessment of the connectivity across the entire area of interest. To this end, a large ROI was created by combining estimates of Brodmann areas (BA) 10 and 11 based on the Brodmann atlas available in MRICron (Rorden et al. 2007). The definition of BA11 here is based on Brodmann (1909) and therefore includes BA12 which was subsequently delineated in Brodmann (1914). This includes areas of the anterior cingulate cortex, including the subgenual cingulate, and extends in an anterior-dorsal direction from the genu to cover the frontal pole. Although the Brodmann atlas available in MRICron cannot be used to determine precise cytoarchitecture, it is sufficient for our current purpose of estimating the gross boundaries of the vmPFC. Binary masks of left BAs 10 and 11 were extracted and combined to form a single vmPFC ROI with their lateral aspects excluded at the level of the superior frontal gyrus. The focus of the investigation was the left vmPFC due to the greater involvement of the left hemisphere in some of the domains thought to be relevant (particularly verbal semantics; Karolis et al. 2019), as well as some evidence for the specific involvement of left vmPFC in theory of mind processing (Leopold et al. 2012). However, the same conclusions may be obtained from the right vmPFC ROI, the results of which are presented in the Supplementary Materials.

The functional connectivity analyses were all computed in Montreal Neurological Institute (MNI) template space using the vmPFC region as a mask. For the structural connectivity analysis, this seed ROI was transformed into native space using the FMRIB's Linear Image Registration Tool (FLIRT) and FMRIB's Nonlinear Image Registration Tool (FNIRT) tools in FSL (FMRIB Software Library; Jenkinson et al. 2001; Jenkinson et al. 2002; Andersson et al. 2010). The ROI for each individual participant was then masked by the individual’s gray matter–white matter interface. While tractography within white matter has been shown to have good validity, tracking within gray matter is unreliable due to the isotropic nature of the diffusion, which causes streamlines to proceed at random until a region of anisotropy (white matter) is reached (Thomas et al. 2014; Reveley et al. 2015; Seehaus et al. 2015). Therefore, in order to increase the reliability of the results obtained, tractography was only performed from the part of the ROI that overlapped with the participant’s gray matter-white matter interface (Saur et al. 2008; Bajada et al. 2017a; Bajada et al. 2017b). In addition, including only those voxels at the cortical boundary minimized the tracking from white matter voxels which may have passed under the cortical surface without connecting to it. To create the final gray-white interface seed ROIs used for tracking, each participant’s T_1_-weighted image was first skull stripped using the brain extraction tool provided in FSL (Smith 2002), co-registered to their B0 image, and segmented using FSL FMRIB's Automated Segmentation Tool (FAST) (Zhang et al. 2001). This interface was then identified in each individual by inputting the resulting white matter mask to an in-house Matlab script which identified the edge of the white matter (i.e., voxels that were white matter but neighboured voxels that were not). To ensure these voxels were located on the interface of gray and white matter and not within the cerebrospinal fluid (CSF), the CSF mask was dilated and areas where it overlapped with the edge of the white matter were removed. The resulting gray matter-white matter interface was used to mask the vmPFC ROI in individual space, and this image was used for tracking. The same process was used to construct a template ROI for the group analysis using the MNI template image. The final ROIs used for the first three participants and the group assessment are displayed in Supplementary Fig. 1.

**Figure 2:**
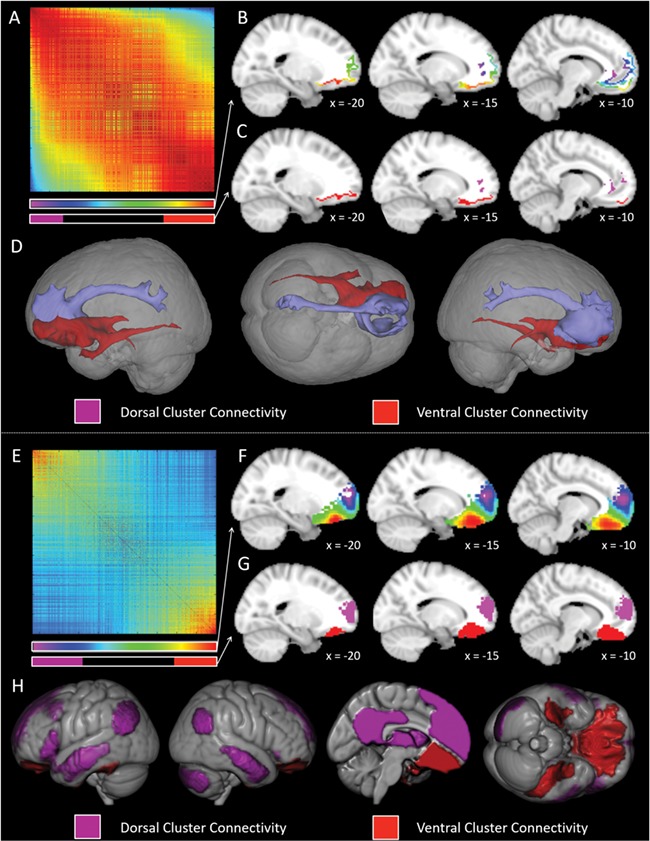
The graded change in structural and functional connectivity across the left ventromedial prefrontal cortex. A. The spectrally reordered similarity matrix of the structural connectivity of each vmPFC voxel. Both rows and columns represent each voxel in the ROI. The voxels with similar connectivity patterns are forced closer together by the spectral-reordering algorithm. The spectrum color bar shown below the matrix represents the correspondence between the position of a voxel on this matrix and the projection of this position on to the brain in B. The purple and red bars represent correspondence between the matrix and the hard cluster location identified in C. B. The projection of each voxels position in the matrix on to the cortex (see A for the correspondence between the color and the matrix). The structural connectivity patterns of the red voxels are most distinct from those of the purple voxels. C. The two distinct hard clusters identified in the matrix projected on to the cortex. The purple and red clusters correspond to either end of the matrix (extent shown by the color bar in A) and therefore have distinct connectivity. D. The connectivity of the red and purple clusters. The connectivity maps of all the voxels identified in each of the two clusters are averaged and thresholded at a low value to remove unlikely connections. The areas and tracts involved in the two clusters appear highly distinct. E. The spectrally reordered similarity matrix of the functional connectivity of each vmPFC voxel. Both rows and columns represent each voxel of the ROI. Voxels with a similar time series are forced closer together by the spectral-reordering algorithm. The spectrum color bar shown below the matrix represents the correspondence between this matrix and the projection of this position on to the brain in F. The purple and red bars represent correspondence between the matrix and the hard cluster location identified in G. F. The projection of each voxels position in the matrix on to the cortex (see A for the correspondence between the color and the matrix). The functional connectivity patterns of the red voxels are most distinct from those of the purple voxels. G. The two distinct hard clusters identified in the matrix projected on to the cortex. The purple and red clusters correspond to either end of the matrix (extent shown by the color bar in E) and therefore have distinct connectivity. H. The distinct, strong connectivity of the red and purple clusters. Each cluster was used as an ROI to estimate its functional connectivity map and the difference in connectivity between the clusters determined through direct comparison of the two maps. The result of this between t-test was masked by the significant connectivity of each cluster determined using a within ROI t-test. Both t-tests were thresholded at a voxel-level threshold of 0.001 and FWE-corrected at the cluster level with a critical cluster level of 0.05.

### Structural Connectivity

#### Image Acquisition

The diffusion-weighted images used were identical to Jackson et al. (2016), Cloutman et al. (2012) and Binney et al. (2012). In brief, a Phillips Achieva 3.0 T system with an eight-element SENSE coil was used to collect a pulsed gradient spin-echo echo planar imaging sequence (TE = 59 ms, TR ≈ 11 884 ms (cardiac gated using peripheral pulse monitor placed upon participant’s index finger [n = 21] or electrocardiograph [n = 3]), G = 62 mT m^−1^, half scan factor = 0.679, 112 × 112 image matrix reconstructed to 128 × 128 using zero padding, reconstructed in-plane voxel resolution 1.875 × 1.875 mm^2^, slice thickness 2.1 mm, 60 contiguous slices, 61 non-collinear diffusion sensitization directions at b = 1200 s/mm^2^ (Δ = 29.8 ms, δ = 13.1 ms), one at b = 0, SENSE acceleration factor = 2.5). In order to minimize distortion effects due to magnetic field inhomogeneties in brain regions near air-tissue boundaries (including vmPFC and anterior temporal areas—both directly relevant to the target of the current study), two volumes were collected for each diffusion gradient direction with opposite phase encoding directions, followed by the application of a distortion correction procedure (Embleton et al. 2010; Binney et al. 2012; Cloutman et al. 2012). A qualitative indication of the accuracy of this procedure was obtained using a co-localized T_2_-weighted turbo spin echo scan with an in-plane resolution of 0.94x0.94 mm, and slice thickness of 2.1 mm. A high resolution structural T_1_-weighted 3D turbo field echo inversion recovery scan (TR ≈ 2000 ms, TE = 3.9 ms, TI = 1150 ms, flip angle eight °, 256 × 205 image matrix reconstructed to 256 × 256, reconstructed in-plane voxel resolution 0.938 × 0.938 mm^2^, slice thickness 0.9 mm, 160 contiguous slices, SENSE factor = 2.5), was acquired to provide precise anatomical information for each participant.

#### Tractography

Tractography was performed for each voxel of the ROI that overlapped with the individual’s gray matter–white matter interface (as performed in Saur et al. 2008; Bajada et al. 2017a; Bajada et al. 2017b). Unconstrained probabilistic tractography was performed using the probabilistic index of connectivity (PICo) algorithm (Parker et al. 2003; Parker et al. 2005). This algorithm samples the orientation of probability density functions (PDFs) across the brain, generated via constrained spherical deconvolution (Tournier et al. 2008) and model-based residual bootstrapping (Haroon et al. 2009; Jeurissen et al. 2011). These methods are better than deterministic methods at dealing with complex fiber orientations, including branching and crossing fibers, and provide a measure of the uncertainty of the resulting connectivity (Jones 2008; Jones 2011). From each voxel within the ROI, 10000 streamlines were propagated, with step size of 0.5 mm. The streamline stopping criteria included reaching a path length greater than 500 mm, or achieving a curvature greater than 180 degrees within a voxel. The tractographic connectivity maps resulting from the seed-based mPFC analyses were thresholded in native space to include only voxels reached by >5 streamlines. These thresholded maps were then normalized to MNI template space using the FLIRT and FNIRT tools in FSL (Jenkinson et al. 2001; Jenkinson et al. 2002; Andersson et al. 2010), for subsequent analyses. Individual participant’s seed voxels were projected on to the template ROI on a nearest neighbor basis. This resulted in one tractography map associated with each voxel in the template ROI for each participant.

***Determining the Voxel-Based Structural Connectivity Gradient and Distinct Subregions.***

All assessments of the structural connectivity of the vmPFC were performed in an identical manner to Bajada et al., (2017b), which was based on the methods presented in Johansen-Berg et al., (2004). All analyses were performed in group space in order for a single gradient map to be constructed. The gradient of structural connectivity across the vmPFC was determined in MNI space. A group ROI template was first created by identifying the gray matter-white matter interface on the MNI template brain and masking this with the left vmPFC ROI. Individual participant’s seed voxels (in MNI space) were then projected onto the template ROI on a nearest neighbor basis. This resulted in each voxel in the template ROI being associated with one tractography map per participant. All tractography maps associated with a particular seed voxel were downsized by a factor of two in each dimension, binarised (at a low threshold of five streamlines reaching the voxel in order to remove spurious connections) and averaged. This resulted in a group tractography map for each voxel in the vmPFC ROI which reflected the proportion of individuals where this connection was identified. A similarity matrix, which represented the connective similarity between each voxel within the vmPFC ROI, was generated using the cosine of the angle between the group tractography images (strung out as vectors) for each pair of voxels. As the structural connectivity values are necessarily positive, the cosine vectors will all be positive, allowing this assumption of spectral-reordering to be met. Spectral-reordering was then used to force voxels with similar connectivity profiles to be closer together in this group similarity matrix (Johansen-Berg et al. 2004; Devlin et al. 2006). The graph Laplacian was constructed as the degree matrix minus the cosine adjacency matrix, following the method outlined in (Johansen-Berg et al. 2004). This is equivalent to solving the generalized eigenvalue problem on a non-normalized Laplacian. The position on this reordered matrix was then back-projected onto the brain in order to display the gradient of similarity of the seed voxels across the vmPFC. Position on this matrix (and therefore similarity of connectivity) was displayed on the brain using a color spectrum (from red to purple). Each voxel within the seed VOI was attributed a value corresponding to its rank in the matrix, e.g., the first voxel in the re-ordered matrix has the value 1. This meant, for instance, that the voxels colored red showed similar connectivity and were most distinct from those colored violet. A more detailed description of this method is provided in Bajada et al. (2017b). The voxels are embedded within a single dimension that is optimal for clustering (Kim et al. 2008). Restricting the solution to a single dimension also allows an intuitive understanding of the main changes in connectivity in an area (Cerliani et al. 2012; Margulies et al. 2016). As spectral reordering merely determines the similarity between each voxels connectivity pattern and orders the voxels by this similarity, it can identify any connectivity gradient from distinct hard clusters (as in Johansen-Berg et al. 2004) to relatively graded changes (as in Bajada et al. 2017b). Therefore, in order to assess how graded the connectivity change across the vmPFC is, we applied a quantitative measure of gradation using an in-house Matlab script (following Bajada et al. 2017b; Jackson et al. 2018). The second smallest eigenvalue of the Laplacian of the similarity matrix ($\lambda_{2} $) reflects the algebraic connectivity of the graph and approaches 0 in a highly clustered matrix (i.e., a matrix with strong similarity within, but weak similarity between clusters), whereas higher values reflect greater gradation (de Abreu 2007). As construction of a connected graph was not enforced, this value can be 0 if the matrix is highly clustered. This is not a statistical test of gradation but simply provides a description of how graded the change in similarity values are, on a scale of near 0 to the value of the largest eigenvalue (typically around 1). Whilst the precise value could be affected by local gradation artificially induced by preprocessing steps, a value much higher than 0 and closer to the value of the largest eigenvalue would reflect a high level of gradation, not likely to be caused by these local changes alone. The $\lambda_{2} $ value was determined for the group matrix. However, combining participants’ data may artificially inflate the level of gradation from that seen in any individual participant. Therefore, the gradation was also assessed in each participant, by spectrally reordering each individual’s matrix (in MNI space) and calculating $\lambda_{2} $ per participant.

In order to identify the regions that had distinct connectivity and determine the connectivity change underlying this transition, we complemented the gradient map with the extraction of hard clusters determined through visual assessment of the ordered group similarity matrix (Johansen-Berg et al. 2004; Bajada et al. 2017b; Jackson et al. 2018). Standard automated approaches are not well-suited to identification of the regions with consistent connectivity alone, whilst avoiding the regions of intermediate connectivity. By identifying these regions visually we can limit the hard clusters to more conservative estimates of the core regions with distinct functional connectivity. This semi-automated approach has been shown to have high cross-validity with other parcellation approaches and individual cytoarchitectural assessment (Klein et al. 2007). The voxels that formed a cluster within a chosen area of the matrix were then back-projected on to the brain and a binary image created to identify the location of each cluster delineated. The connectivity of these hard clusters was then examined and compared to enable greater understanding of the precise changes in connectivity patterns. This connectivity was computed as the average of the (non-binarised) tractography maps of all the voxels identified within a hard cluster. Thus, the resulting map reflects the group average connectivity (i.e., average number of streamlines) of a voxel within the cluster. As this is fairly stringent (by reflecting the average of each voxel and not the sum of all the voxels within the cluster) this group map is shown at a minimal threshold of five streamlines reaching a voxel in order to remove spurious connections. By identifying both the gradient of change as well as performing a hard parcellation, we were able to examine the vmPFC structural connectivity for both transitional zones and distinct clusters.

**Figure 3:**
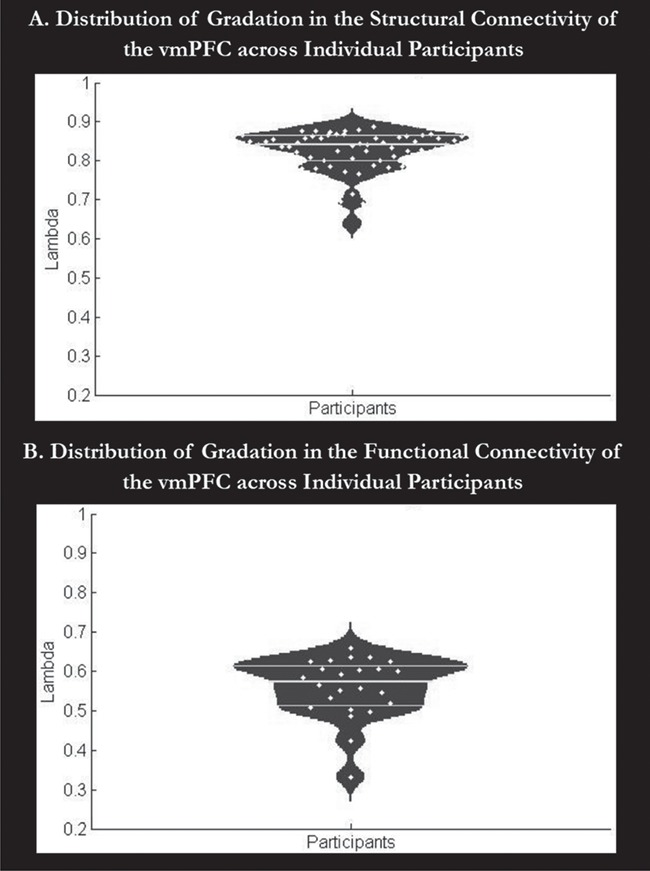
The distribution of the individuals’ gradation metric values (lambda). A. The distribution of the lambda values obtained per participant in the structural connectivity assessment. B. The distribution of the lambda values obtained per participant in the functional connectivity assessment. The horizontal lines show the 25^th^ and 75^th^ percentiles and the mean lambda values. Values of lambda near 0 reflect hard clusters, whereas higher numbers reflect a graded change in connectivity. Participants consistently show graded changes in the functional and structural connectivity of their vmPFC.

### Functional Connectivity

#### Image Acquisition and Preprocessing

In order to preserve signal within regions of high magnetic susceptibility including the critical vmPFC region, resting-state scans acquired using a dual-echo gradient echo echo planar imaging were used. Both a short echo (12 ms) and a standard long echo (35 ms) are acquired in parallel and then linearly combined. This reduces signal loss in inferior frontal and temporal cortices where signal dropout is high, without a loss of signal elsewhere in the brain (Poser et al. 2006; Halai et al. 2014). Ghosting artifacts were reduced through the use of a 45 ° tilt off the anterior commissure-posterior commissure line. The resting-state data acquisition method has been described previously (Jackson et al. 2016; Jung et al. 2016; Jackson et al. 2018). Participants were asked to lie still and fixate on a cross (Van Dijk et al. 2012) whilst wearing noise-canceling Mk II headphones (MR Confon). A total of 130 volumes were collected over 6.25 min with whole brain coverage (240 x 240 mm, resolution matrix 80 x80, voxel size three x three x four mm). A Phillips Achieve 3.0 T system with a 32 channel SENSE coil (sense factor 2.5) was used. The TR was 2.8. A T1-weighted structural scan (in-plane resolution 0.938; slice thickness 1.173) was obtained for anatomical reference.

Statistical parametric mapping (SPM 8) software (Wellcome Trust Centre for Neuroimaging) was used for slice timing correction, realignment and coregistration to the individual’s structural image. Resting-state connectivity analyses are highly susceptible to motion artifact which can be reduced through appropriate preprocessing methods (Anderson et al. 2011; Power et al. 2014; Power et al. 2015). Preprocessing was identical to a prior functional parcellation of the temporal cortex (Jackson et al. 2018), as well as prior seed-based functional connectivity analyses (Jackson et al. 2016), both employing the same dataset. This included the use of four methods shown to greatly reduce the effects of motion: censoring; global signal regression; 24 motion parameter regression; and scrubbing of high motion time points. The Data Processing Assistant for Resting State fMRI Advanced Edition (DPARSFA, V2.3) toolbox (Chao-Gan et al. 2010) was used to regress out nuisance covariates and normalize and smooth (to an eight mm full-width half maximum Gaussian kernel) the images using DARTEL (Ashburner 2007). Time points with high motion artifact (determined as greater than one mm translation or a z-score signal deviation from the mean of greater than 2.5) were identified using the ARtifact detection Tools software package (ART; w.nitrc.org/projects/artifact_detect). These time points were included as nuisance covariates alongside 24 motion parameters (created from the standard six parameters using a Volterra expansion; Friston et al. 1996) and the white matter, CSF and global tissue signals. Censoring participants with less than five minutes of data after regression of nuisance time points or a single translation of more than three mm resulted in the exclusion of six participants; therefore analyses were performed on 71 participants. These methods are consistent with prior resting-state studies (Weissenbacher et al. 2009; Anderson et al. 2011; Van Dijk et al. 2012; Yan et al. 2013; Power et al. 2014; Power et al. 2015; Jackson et al. 2016) and assessment in Jackson et al., (2016) suggested motion artifact removal was successful.

#### Determining the Voxel-Based Functional Connectivity Gradient and Distinct Subregions

The gradient of functional connectivity of the vmPFC was determined using the Matlab-based graphical user interface (referred to as Functional Parcenip) provided in Jackson et al., (2018). This employed a spectral-reordering method, analogous to that of the structural connectivity analysis described here, that has previously been used to show graded changes and distinct clusters within the temporal cortex (Jackson et al. 2018). All functional connectivity assessments were performed in an identical manner to Jackson et al., (2018). As with the structural analysis, this technique was chosen to allow identification of graded connectivity changes and areas of distinct connectivity. Although there may be artefactual causes of local gradients in the functional data (e.g., smoothing, interpolation and motion artifacts), the underlying activity may also be graded and it may not be optimal to force each voxel in to hard clusters. Reciprocally, determining hard clusters within the graded result allows the connectivity of each to be analyzed, enabling interpretation of the connectivity change.

All analyses were performed within MNI space to allow simple transition from individual to group data. The time series of every voxel within the vmPFC ROI was extracted. Then a cosine similarity matrix of the time series of each voxel per individual was created. As per Johansen-Berg et al. (2004), a value of one was added to every value in this matrix in order to ensure that all values were positive and thus, meet the criteria for performing spectral reordering. This simply rescales the matrix, maintaining the ordering of connections. Negative relationships have the lowest values, followed by no relationship and then positive relationships. This matrix was z-score normalized to allow each participant’s result to be averaged (Dunlap et al. 1983), creating a group average similarity matrix. This matrix was then transformed back from z-scores to cosine values. Each value in this matrix represented the average similarity over time of two vmPFC voxels at a group level, i.e., their functional connectivity. This matrix was then spectrally reordered so that vmPFC voxels that were more similar in their time course were moved closer together within the matrix. The graph Laplacian was constructed as the degree matrix of the cosine adjacency matrix, following the method outlined in (Johansen-Berg et al. 2004). This is equivalent to solving the generalized eigenvalue problem on a non-normalized Laplacian. The resulting gradient of connectivity was visualized on the brain through back-projection using a color spectrum from red to purple (therefore voxels with a similar time course were represented by a similar color). The level of gradation in the group matrix was evaluated by calculating $\lambda_{2} $ with an in-house Matlab script, as in the structural connectivity analyses and prior research (Bajada et al. 2017b; Jackson et al. 2018). Additionally, the individual participant’s matrices were spectrally reordered and $\lambda_{2} $ determined for each. This gave an estimate of the average gradation within a participant, without artificially inducing gradation due to individual differences. Hard clusters were determined through visual inspection of the reordered group matrix and back-projected as binary values onto the brain. All of these steps were performed using the Functional Parcenip graphical user interface. The functional connectivity differences, that had driven the separation between these clusters, were then estimated by entering these binarised hard clusters into a functional connectivity analysis within DPARSFA. In order to assess the difference in connectivity over the ROI (which may be subtler than whole-brain differences), a between t-test was computed in SPM8 (Wellcome Trust Centre for Neuroimaging) to identify regions showing greater functional connectivity to one hard cluster than the other. In addition, this differential connectivity may be constrained by the significant connectivity of each cluster to understand which regions are both a) strongly connected to that cluster and b) display differential connectivity with the two vmPFC regions. This allows identification of the connectivity patterns central to splitting these regions and determining their potential functions. Therefore, the results of the between ROI t-test were constrained by the significant (voxel level P < 0.001, cluster level P < 0.05) results of a within t-test performed in SPM8 for each cluster. For a deeper understanding of how this image is created, the result of each individual t-test is provided in the Supplementary Materials.

## Results

### The Voxel-Based Structural Connectivity Gradient and Distinct Subregions

The results of the structural connectivity-based assessment of the left vmPFC are presented in Figure 2. The right hemisphere results are similar and are presented in Supplementary Fig. 2 and Supplementary Table 1. The spectrally-reordered similarity matrix showing the graded change in structural connectivity across voxels in the vmPFC region is shown in Fig. 2A. The rank order in which the seed voxels are positioned in this matrix reflects the similarity of their connections. This positioning of the voxels within the matrix was visually coded on the brain using a color spectrum from red (left) to purple (right), to reveal the pattern of change in connectivity across the vmPFC (Fig. 2B). The maps of this result (in the left and right vmPFC) are available for visualization or download on NeuroVault (Gorgolewski et al. 2015) at https://neurovault.org/collections/4798/. From this, it can be seen that the structural connectivity of the vmPFC is principally organized along a dorsomedial-lateroventral axis. This results in a change in connectivity from the medial wall (particularly perigenual and dorsal regions) towards the basal surface of the vmPFC (i.e., the medial orbitofrontal cortex). The quantitative measure of gradation was not close to 0 (reflecting distinct hard clusters), instead $\lambda_{2} $ equalled 0.9207. This indicates an extremely high degree of gradation, indicating that a simple hard split (including all voxels) would not represent the data well. This is supported by assessment of the $\lambda_{2} $ of each individual participant’s matrix. The distribution of the individuals’ $\lambda_{2} $ values are shown in Figure 3A. The average individual $\lambda_{2} $ was 0.5589 (SD = 0.0756, 25^th^ percentile = 0.5168, 75^th^ percentile = 0.6106). Although this is lower than the group estimate, it is not near 0 and reflects a high level of individual gradation similar to the group estimate of gradation in the temporal lobe (Bajada et al. 2017b). The first ten individual’s gradient maps are displayed in Supplementary Fig. 3. However, visual inspection of the spectrally-reordered group similarity matrix revealed that while there is a graduation of connective change across the vmPFC, the two regions identified as most distinct from each other (i.e., at opposite, red versus purple, ends of the matrix) appeared to be associated with distinct clusters (represented by the red and purple color bars underneath the matrix). The brain areas corresponding to these two clusters were individually extracted and projected onto the brain (Fig. 2C), which showed that the cluster in red corresponded well to the medial orbitofrontal cortex (mOFC), whereas the purple cluster was focussed on regions of the medial wall more dorsal than the OFC and which particularly included areas near the genu (in the dorsal-ventral and anterior-posterior directions). Although not labelled cingulate cortex in the Brodmann atlas used to determine the vmPFC ROI, this cluster also appears to partially overlap anterior cingulate cortex. Comparison with the atlas estimates of Brodmann areas indicates that the entire mOFC cluster is within BA 11 whereas the medial wall cluster appears to include both BA 10 and BA 11.

The structural connectivity of the two clusters was then determined in order to identify the specific connectivity change across the vmPFC that drove this parcellation. For each cluster, the average structural connectivity map of each constituent voxel is displayed (see Fig. 2D). The purple ‘medial wall’ cluster was found to be connected to anterior, mid and posterior cingulate cortex and the precuneus. Although it is not possible to definitively identify the specific tract underlying this connectivity, this connective pathway in an anterior-posterior direction across the cingulate is highly consistent with the cingulum (Catani et al. 2002). The red mOFC cluster was found to be connected to anterior and posterior temporal cortices, medial temporal cortices including the hippocampus and the amygdala, as well as the occipital lobe. This pattern of connectivity is consistent with the contribution of both the inferior fronto-occipital fasciculus (IFOF) and the uncinate fasciculus (UF). Whilst both of these tracts have frontal connections, the IFOF terminates in the ventral occipital cortex whereas the UF connects to superior anterior temporal cortex (Gloor 1997; Catani et al. 2002; Catani et al. 2008; Martino et al. 2010; Bajada et al. 2015). This examination of the white matter tracts underlying the two vmPFC clusters reveals that the structural connectivity of the vmPFC varies in a graded fashion, transitioning from connectivity via the cingulate to posterior cingulate cortex to connectivity via the UF and IFOF to anterior, posterior and medial temporal cortices and the occipital lobe.

### The Voxel-Based Functional Connectivity Gradient and Distinct Subregions

To support the structural connectivity analyses and help determine the involvement of subregions of the vmPFC in functional networks, the gradient of functional connectivity of the vmPFC was independently determined. The spectrally-reordered similarity matrix of the time course of each vmPFC voxel is presented in Fig. 2E. The position of each seed voxel in this matrix was shown on the cortex using a color spectrum from red to purple (as with the structural connectivity; Fig. 2F). The maps of this result (in the left and right vmPFC) are available for visualization or download on NeuroVault (Gorgolewski et al. 2015) at https://neurovault.org/collections/4798/. Visual examination of these results reveals that the pattern of connectivity change appears less graded than that found for the structural connectivity assessment. The quantitative measure of gradation is somewhat lower than for the structural connectivity results, at 0.8875. However, this still reflects an extremely high level of gradation. Indeed, the assessment of gradation for each participant results in a higher $\lambda_{2} $ on average (mean = 0.8263, SD = 0.0563, 25^th^ percentile = 0.8005, 75^th^ percentile = 0.6190) as shown in Fig. 3B. This confirms a strong graded change in functional connectivity across the vmPFC. The first ten individual’s gradient maps are displayed in Supplementary Fig. 4. These differences may reflect real variation between the functional and structural connective patterns of the vmPFC, or an artefactual difference due to preprocessing factors or methodological dissimilarities (see the Discussion for a consideration for the relationship between the functional and structural connectivity-based results). Regardless of the exact slope of the gradient, both the structural and functional connectivity analyses show a gradient with the same principal direction along a dorsomedial-lateroventral axis, with relatively lateral mOFC being most distinct from areas of the dorsal medial wall.

Despite the graded connectivity changes, visual inspection of the reordered matrix clearly revealed that there were two distinct clusters in the functional data, shown in Fig. 2G in red and purple (red and purple color bars in Fig. 2E show the correspondence of these clusters with the matrix). The red cluster is located in the mOFC, and corresponds well to the structural connectivity analysis. The purple cluster is focussed around the dorsal tip of the vmPFC ROI, extending ventrally to the approximate height of the genu. The purple clusters identified in the functional and structural parcellations demonstrate partial overlap, with both including areas of the medial wall. However, the focus of the functional cluster is more dorsal than the structural connectivity-based cluster.

The regions that were both significantly functionally connected to a cluster and showed significantly greater connectivity to that cluster than the other were determined. These areas are displayed in Fig. 2H, with a voxel-level threshold of 0.001 and a family-wise error (FWE) correction at the cluster level with a critical cluster level of 0.05. Detailed results are shown in Table 1. To review all connections to each cluster, and all relative differences in connectivity, consult Supplementary Fig. 5 and Supplementary Table 2 & Table 3. Due to the position of the two vmPFC clusters it is not surprising that the dorsal (purple) cluster showed greater connectivity with the dorsal mPFC and the ventral (red) cluster with the most basal mPFC (i.e., across the OFC). First looking at the purple cluster, this medial wall region showed greater connectivity to anterior and posterior cingulate cortex and the precuneus, as well as the angular gyrus, lateral anterior temporal cortex, inferior frontal gyrus, posterior region of the parahippocampal gyrus, cerebellum and thalamus. Overall, the regions showing greater functional connectivity to the purple cluster correspond to the regions of the DMN.

**Table 1:** Differential, significant functional connections of the two core distinct vmPFC clusters identified in the functional resting-state data

Contrast	Region of Activation	Cluster extent (voxels)	Max z value	P value (FWE corrected)	Peak MNI Coordinate		
					X	Y	Z
Purple (Dorsal) Cluster > Red (Ventral) Cluster	Bilateral dmPFC, ACC, IFG, precuneus, PCC, thalamus & L pMTL, sTP & MTG	9207	Inf	>.001	−3	57	15
	L AG	703	Inf	>.001	−51	−60	30
	Cerebellum	671	Inf	>.001	27	−81	−36
	R AG	366	Inf	>.001	54	−57	30
	R MTG	632	6.77	>.001	63	−12	−15
Red (Ventral) Cluster > Purple (Dorsal) Cluster	Bilateral OFC & aPHG	2435	Inf	>.001	−12	36	−21
	L vATL & pITC	404	Inf	>.001	−51	−39	−27
	R vATL	180	6.73	.002	33	−27	−30

Clusters significant at 0.05 after FWE correction. L = left, R = right, a = anterior, p = posterior, dmPFC = dorsomedial prefrontal cortex, IFG = inferior frontal gyrus, PCC = posterior cingulate cortex, MTL = medial temporal lobe, sTP = superior temporal pole, MTG = middle temporal gyrus, AG = angular gyrus, OFC = orbitofrontal cortex, vATL = ventral anterior temporal lobe, PHG = parahippocampal gyrus and ITC = inferior temporal cortex.

Looking at the red cluster, this mOFC region showed greater connectivity to inferior temporal cortices, ventral anterior temporal lobe and a small anterior parahippocampal gyrus region. The visual ventral route includes both the inferior temporal cortices and the ventral anterior temporal cortex. However, this ventral anterior region is not merely visual, but is crucial for multimodal semantic cognition (see Discussion). These areas align well with the structural connectivity of the ventral vmPFC cluster and may be critical for understanding the role of this region. These results indicate that the purple (medial wall) and red (mOFC) clusters identified show distinct functional connectivity, with the connectivity profile across the vmPFC reflecting a gradient from DMN to sensorimotor and multimodal semantic regions.

## Discussion

The structural and functional connectivity of each voxel in the vmPFC was assessed to delineate changes in the pattern of connectivity across this region and identify possible subregions with distinct connectivity profiles. A highly graded change in both the structural and functional connectivity of the vmPFC was identified. However, regions with distinct connectivity could be distinguished at either end of this gradient. Therefore, the vmPFC displays a graded transition between two areas of distinct connectivity. Both structural and functional connectivity were varied along a dorsomedial to ventrolateral axis, from relatively dorsal regions of the medial wall to the basal surface of the mPFC. The structural connectivity varied from cingulate connections (to the posterior cingulate cortex and precuneus) at one end of the axis, to UF and IFOF connections (to anterior, posterior and medial temporal cortices and the occipital lobe) at the other. The functional connectivity changes along the same axis highlighted the differential involvement of well-known resting state networks across the vmPFC. The dorsomedial end of the axis showed greater connectivity throughout the DMN, including the structurally connected cingulate cortex and precuneus, as well as the angular gyrus, lateral anterior temporal lobe (ATL), inferior frontal gyrus and medial temporal lobe (Buckner et al. 2008; Greicius et al. 2009). The ventrolateral end of the axis showed greater functionally connectivity with the structurally connected ventral visual stream and multimodal regions that receive direct input from this processing route (the ventral aspects of the anterior portion of the temporal lobe and the medial temporal lobe). Therefore, the vmPFC shows a graded change in connectivity from a DMN-connected medial wall area to a distinct mOFC region associated with the visual stream and the multimodal ventral ATL. The potential functional roles of the mOFC and medial wall areas are considered.

The change in connectivity across the vmPFC was found to be relatively graded, including an area of intermediate connectivity between the two distinct regions. Spectral reordering approaches do not change the nature of the similarity within the data and can therefore, show any level of gradation that exists in the connectivity pattern of a region. The spectral-reordering approach employed here, has previously been used to demonstrate both graded (Bajada et al. 2017b; Haak et al. 2018) and hard parcellations (Johansen-Berg et al. 2004) within the brain and does not presuppose the nature of the connectivity change. Instead this graded change is a direct reflection of the data. It may be of interest, therefore, to contrast the current result to the small number of prior studies that have attempted a hard parcellation of some or all of the vmPFC based on its connectivity or co-activation (Kahnt et al. 2012; Liu et al. 2013; Moayedi et al. 2015; Ray et al. 2015; de la Vega et al. 2016; Fan et al. 2016; Thiebaut de Schotten et al. 2016). The vmPFC parcellation studies have identified distinct dorsal and ventral regions separated around the level of the genu (Liu et al. 2013; Ray et al. 2015; de la Vega et al. 2016; Fan et al. 2016; Thiebaut de Schotten et al. 2016). This hard split may well reflect the same graded ventrolateral-dorsomedial gradient of connectivity determined here, with distinct connectivity between OFC and more dorsal medial wall regions. However, unlike assessing the full change in connectivity pattern, these hard parcellations are unable to separate the core regions with distinct connectivity, from the intermediate regions whose connectivity shows a graded transition between the different profiles. Therefore, the core difference between the OFC and the medial wall is obscured, as well as the underlying connectivity difference. Determining the core subregions and excluding intermediate areas may improve the specificity of the correspondence with function, which is often low (de la Vega et al. 2016; Thiebaut de Schotten et al. 2016). Higher order cognitive regions such as the vmPFC are known to have a complex relation to function, with overlap between regions involved in different functional networks (Hutchison et al. 2013; Jackson et al. 2019). One caveat of the current approach is that the change in connectivity is embedded within a single (optimal) dimension (Kim et al. 2008). This allows the main connectivity differences in an area to be determined whilst maintaining interpretability of the results, however fine-grained detail may be missed. Focusing on the large changes across the region is consistent with our aim and prior analyses of gradients in the brain (Cerliani et al. 2012; Margulies et al. 2016; Bajada et al. 2017b; Jackson et al. 2018).

The gradients of functional and structural connectivity had a high level of convergence, with both showing gradation along the same principal axis. This corresponds well to the small number of prior studies where concurrent functional and structural parcellations have been performed, which show a high level of convergence alongside a secondary, smaller level of divergence (Zhang et al. 2010; Wang et al. 2015). Information in different modalities may allow parcellations to capture distinct aspects of functional organization, including organization at differing levels of granularity (Eickhoff et al. 2011; Cloutman and Lambon Ralph 2012; Eickhoff et al. 2018). Therefore, convergence across methods based on different modalities allows greater support for the conclusions drawn. The strong correspondence between the structural and functional connectivity-based assessments of the vmPFC allows interpretation of the underlying gradient as reflective of the functional organization of the vmPFC. Although their relationship is complex, structural connectivity typically leads to functional connectivity, yet the presence of a functional connection does not necessitate a structural connection (Greicius et al. 2009; Honey et al. 2009). Thus, these two types of information may reflect cortical organization at a slightly different level of granularity leading to some level of divergence. For instance, the greater level of gradation in the individual’s structural data may suggest that the graded structural connectivity pattern subserves a slightly less graded pattern of organization in the functional connectivity. Alternatively, any divergence between the two results may simply reflect the various differences in the data sources, artifacts and processing which underlie the different analyses. As the focus of the current investigation is the principal large-scale change in connectivity across the vmPFC, a great focus on the fine scale differences between the two results may be inappropriate. However, some divergence may be seen in the precise location of the dorsal extreme of the two results (located more dorsally in the functional connectivity results and closer to the genu in the structural connectivity results). Understood in the context of the larger convergent results, this small divergence could be due to a ventral subregion of a functionally cohesive area having stronger DMN-associated structural connectivity. Alternatively, it may be due to the greater effect of distance on similarity in the functional connectivity assessments or the spatial differences between the voxels examined in the two assessments. Further explorations of the relations between the organizational gradients in structural and functional data may be informative.

By assessing the full pattern of connectivity across the vmPFC we were able to identify core OFC and medial wall regions with distinct connectivity. The separation of orbitofrontal and medial regions corresponds well to multiple cytoarchitectural schemes in macaque and human brains (see Fig. 1; von Economo et al. 1925; Walker 1940; Sarkisov et al. 1949; Carmichael et al. 1994; Petrides & Pandya 1994; Öngür et al. 2003) and is reminiscent of the classical distinction between medial and orbital networks identified in macaques (Carmichael et al. 1995a, 1995b, 1996; Öngür et al. 1998; Öngür et al. 2000; Kondo et al. 2003, 2005; Klein et al. 2010) and rats (Ray et al. 1992; Ray et al. 1993; Floyd et al. 2000, 2001). In macaques, the medial network shows greater connectivity to motor regions and retrosplenial cortex, whilst the orbital network shows greater connectivity to sensory regions and area TE (the primate homologue of the human ventral ATL). Similar distinctions can also be identified between lateral and medial OFC (Cavada et al. 2000). TE is often considered a higher order visual area yet, damage to TE in primates or the ventral ATL in humans, causes a multimodal semantic impairment (Kluver et al. 1937; Patterson et al. 2007; Lambon Ralph 2014). Neuroimaging, neurostimulation, electrophysiological and intracortical electrode studies support the multimodal semantic role of the ventral ATL (Marinkovic et al. 2003; Matsumoto et al. 2004; Pobric et al. 2007; Binney et al. 2010; Visser et al. 2012; Shimotake et al. 2014; Chen et al. 2016). As such it appears that both human and macaque vmPFC connectivity shifts from sensory and multimodal semantic regions in relatively lateral regions of the mOFC to DMN-related connectivity in more dorsal medial wall regions. This critical distinction between areas connected to the DMN and those associated with semantic cognition areas fits recent functional work distinguishing semantic and default mode networks (Humphreys et al. 2015; Jackson et al. 2019).

The distinction between mOFC and medial wall regions may reflect a difference in function. Whilst the precise function of the OFC remains debated (e.g., Stalnaker et al. 2015), it seems to require the combination of unimodal sensory and affective inputs, as well as multimodal semantic information from the ATL. Indeed, MEG studies and fMRI meta-analyses implicate the vmPFC in semantic cognition (Marinkovic et al. 2003; Pylkkänen et al. 2007; Binder et al. 2009; Noonan et al. 2013). However, activity in the OFC has been shown to be invariant to cue identity, instead reflecting subjective economic value (Padoa-Schioppa et al. 2006;Plassman et al. 2007; Levy et al. 2012). Thus, perhaps it is not semantic cognition per se that is performed here but a post-semantic assessment of context-specific object-reward associations (Murray et al. 2007; Rushworth et al. 2007a; Klein et al. 2010; Roy et al. 2012; Stalnaker et al. 2015). A value-related interpretation of the mOFC is in-keeping with dysfunction following OFC damage (Fellows et al. 2007; Camille et al. 2011; Rudebeck et al. 2011b) and prior hard parcellation analyses, where basal vmPFC regions have been linked to value, reward and food-related cognition (de la Vega et al. 2016; Thiebaut de Schotten et al. 2016). A factor analysis of large-scale meta-analysis results identified a similar split with a ventral vmPFC region involved in emotion, reward and autonomic processing, theorized to relate to the generation of affective meaning (Roy et al. 2012). Alternatively, semantic and affective information could interact dynamically at an early stage of visual object recognition, so that recognition of objects eliciting fear and reward signals can be facilitated to promote fast adaptive responses (Bar 2003).

The medial wall cluster connected to the DMN and corresponds well to the core mPFC DMN region (Greicius et al. 2009). However, the function of the DMN is not well understood. Suggested functions include episodic memory, social cognition, mind-wandering, spontaneous cognition and internally directed attention (Mason et al. 2007; Buckner et al. 2008; Andrews-Hanna et al. 2010; Mars et al. 2012). To date, DMN research has not been successfully integrated into the study of the functional role of the vmPFC due to core methodological differences between research domains (Delgado et al. 2016), as well as a paucity of formal methods to associate resting-state networks and function (Jackson et al. 2019). In addition, hard parcellations of the vmPFC and large-scale meta-analytical techniques have demonstrated a correspondence between relatively dorsal medial regions and social cognition (Roy et al. 2012; de la Vega et al. 2016; Thiebaut de Schotten et al. 2016), and deficits in social processing and theory of mind have been associated with vmPFC damage (Stone et al. 1998; Mah et al. 2005; Shamay-Tsoory et al. 2005). Within a reward-based account of the vmPFC, the medial wall could have a particular role in calculating the overall expected value of actions, particularly social actions, using a single ‘currency’ for decision making (Rushworth et al. 2007b; Klein et al. 2010; Rudebeck et al. 2011a; Euston et al. 2012). By demonstrating the relationship between vmPFC subregions and distinct functional networks we hope to inform the ongoing debate between differing functional perspectives of the vmPFC.

## Supplementary Material

Jackson_supplementary_data_new_bhz079
