# Supplementary material for: The Graded Change in Connectivity across the Ventromedial Prefrontal Cortex Reveals Distinct Subregions
Source: Cereb Cortex. 2019 Apr 26;30(1):165–80. doi: 10.1093/cercor/bhz079 (PMC7029692; doi:10.1093/cercor/bhz079)
Supplement: Jackson_supplementary_data_new_bhz079 [file jackson_supplementary_data_new_bhz079.docx]

The Graded Change in Connectivity across the Ventromedial Prefrontal Cortex Reveals Distinct Subregions

Rebecca L. Jackson, Claude J. Bajada, Matthew A. Lambon Ralph & Lauren L. Cloutman

**Supplementary Materials**


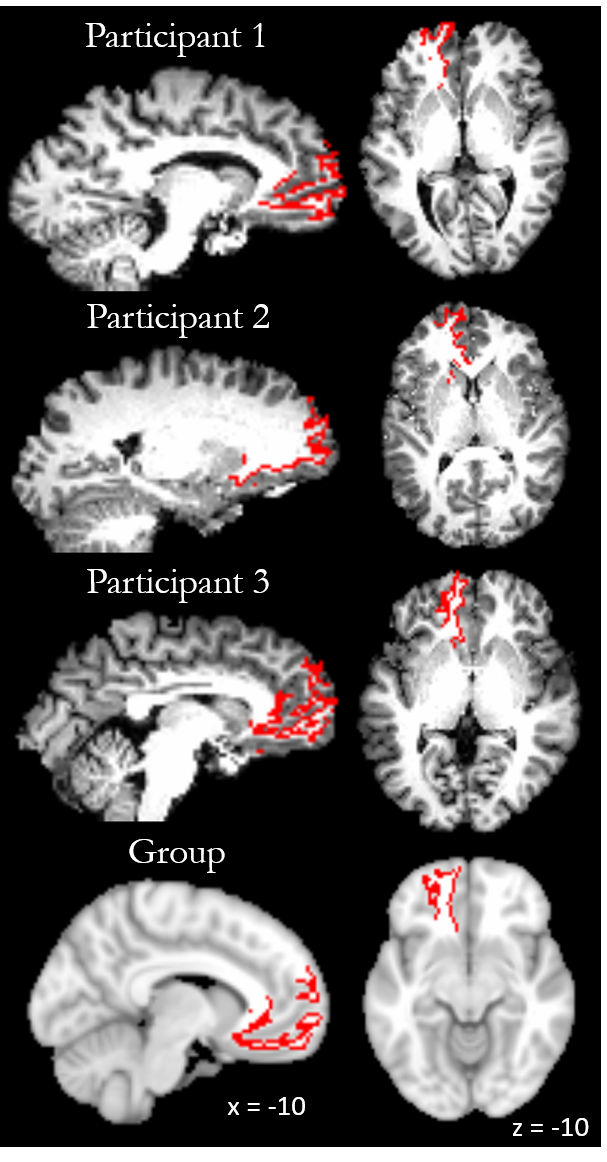


*Supplementary Figure 1.* Example ROIs of the grey-white interface of the left vmPFC in the first three participants and the group ROI used for the structural connectivity assessment.


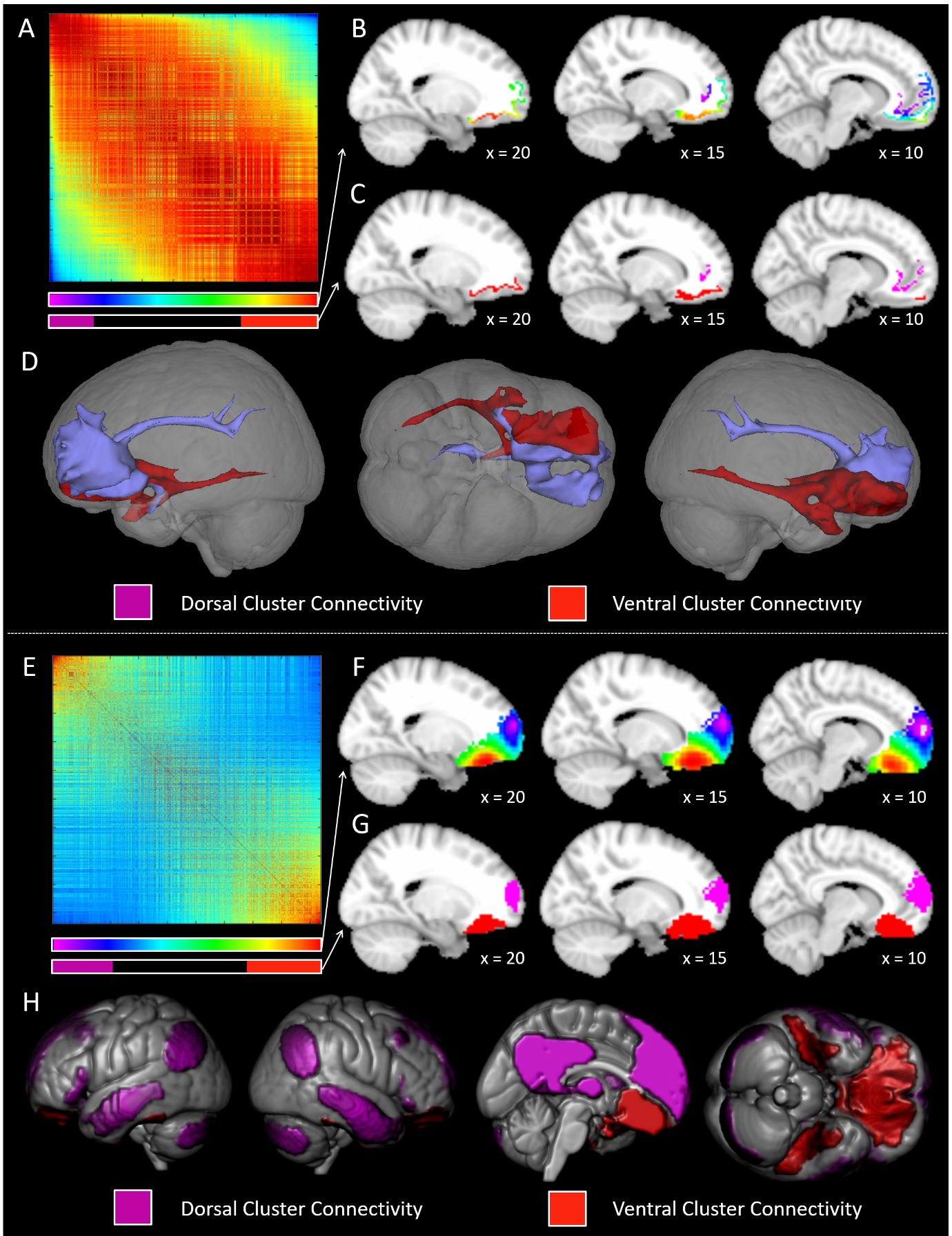


*Supplementary Figure 2.* The graded change in structural and functional connectivity across the right ventromedial prefrontal cortex. A. The spectrally reordered similarity matrix of the structural connectivity of each right vmPFC voxel. Both rows and columns represent each voxel in the ROI. The voxels with similar connectivity patterns are forced closer together by the spectral-reordering algorithm. The spectrum colour bar shown below the matrix represents the correspondence between the position of a voxel on this matrix and the projection of this position on to the brain in B. The purple and red bars represent correspondence between the matrix and the hard cluster location identified in C. B. The projection of each voxels position in the matrix on to the cortex (see A for the correspondence between the colour and the matrix). The structural connectivity patterns of the red voxels are most distinct from those of the purple voxels. C. The two distinct hard clusters identified in the matrix projected on to the cortex. The purple and red clusters correspond to either end of the matrix (extent shown by the colour bar in A) and therefore have distinct connectivity. D. The connectivity of the red and purple clusters. The connectivity maps of all the voxels identified in each of the two clusters are averaged and thresholded at a low value to remove unlikely connections. The areas and tracts involved in the two clusters appear highly distinct. E. The spectrally reordered similarity matrix of the functional connectivity of each right vmPFC voxel. Both rows and columns represent each voxel of the ROI. Voxels with a similar time series are forced closer together by the spectral-reordering algorithm. The spectrum colour bar shown below the matrix represents the correspondence between this matrix and the projection of this position on to the brain in F. The purple and red bars represent correspondence between the matrix and the hard cluster location identified in G. F. The projection of each voxels position in the matrix on to the cortex (see A for the correspondence between the colour and the matrix). The functional connectivity patterns of the red voxels are most distinct from those of the purple voxels. G. The two distinct hard clusters identified in the matrix projected on to the cortex. The purple and red clusters correspond to either end of the matrix (extent shown by the colour bar in E) and therefore have distinct connectivity. H. The distinct, strong connectivity of the red and purple clusters. Each cluster was used as an ROI to estimate its functional connectivity map and the difference in connectivity between the clusters determined through direct comparison of the two maps. The result of this between t-test was masked by the significant connectivity of each cluster determined using a within ROI t-test. Both t-tests were thresholded at a voxel-level threshold of .001 and FWE-corrected at the cluster level with a critical cluster level of .05.

Supplementary Table 1. Differential, significant functional connectivity of the two right vmPFC clusters identified in the hard parcellation of the functional resting-state data.

| Contrast | Region of Activation | Cluster extent (voxels) | Max z value | P value (FWE corrected) | Peak MNI Coordinate | | |  |
| --- | --- | --- | --- | --- | --- | --- | --- | --- |
|  |  |  |  |  | X | Y | Z | |
| Purple (Dorsal) Cluster > Red (Ventral) Cluster | Bilateral dmPFC, ACC, IFG, insula, precuneus, PCC, thalamus, sTP, MTG & aITG | 10200 | Inf | >.001 | 6 | 60 | 12 | |
|  | L AG | 755 | Inf | >.001 | -51 | -60 | 27 | |
|  | R AG | 714 | Inf | >.001 | 54 | -57 | 30 | |
|  | Cerebellum | 427 | Inf | >.001 | -27 | -81 | -36 | |
|  | Cerebellum | 519 | Inf | >.001 | 27 | -81 | -33 | |
|  | L MTL | 148 | 5.36 | .006 | -21 | -21 | -15 | |
| Red (Ventral) Cluster > Purple (Dorsal) Cluster | Bilateral OFC | 2536 | Inf | >.001 | 15 | 33 | -21 | |
|  | L vATL & ITC | 298 | Inf | >.001 | -51 | -42 | -27 | |
|  | R vATL & ITC | 218 | 6.69 | .001 | 33 | -24 | -30 | |

*Clusters significant at .05 after FWE correction. L = left, R = right, a = anterior, p = posterior, dmPFC = dorsomedial prefrontal cortex,* *PCC = posterior cingulate cortex, MTL = medial temporal lobe, sTP = superior temporal pole, MTG = middle temporal gyrus, ITG = inferior temporal gyrus, AG = angular gyrus, OFC = orbitofrontal cortex, vATL = ventral anterior temporal lobe and ITC = inferior temporal cortex.*


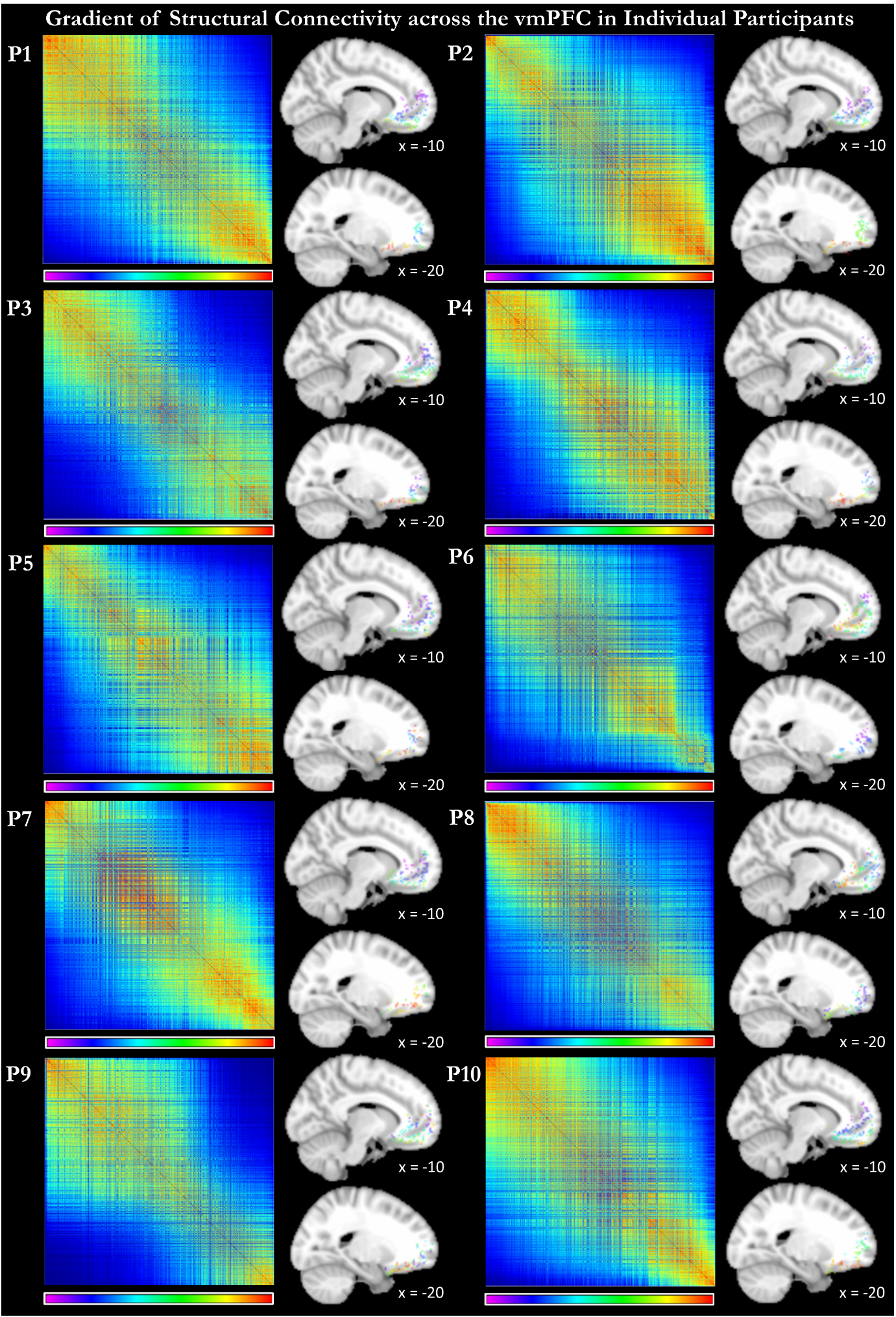


*Supplementary Figure 3.* The graded change in structural connectivity across the left ventromedial prefrontal cortex in the first 10 participants. For each participant the spectrally reordered similarity matrix is shown on the left. Both rows and columns represent each voxel in the ROI. The voxels with similar connectivity patterns are forced closer together by the spectral-reordering algorithm. The spectrum colour bar shown below the matrix represents the correspondence between the position of a voxel on this matrix and the projection of this position on to the brain to the right of the matrix. The brain images display the projection of each voxels position in the matrix on to the cortex. The structural connectivity patterns of the red voxels are most distinct from those of the purple voxels.


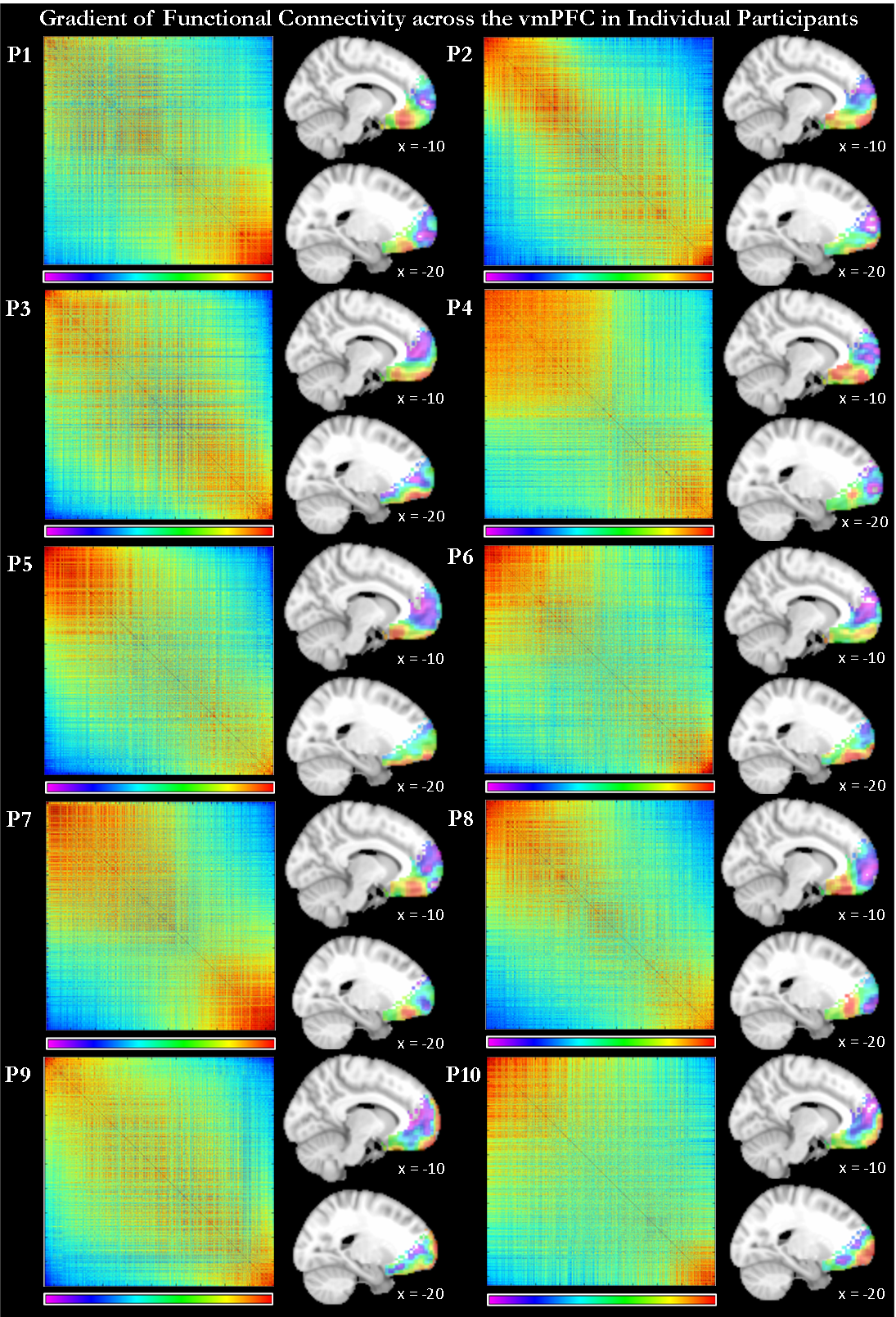


*Supplementary Figure 4.* The graded change in functional connectivity across the left ventromedial prefrontal cortex in the first 10 participants. For each participant the spectrally reordered similarity matrix is shown on the left. Both rows and columns represent each voxel in the ROI. The voxels with similar connectivity patterns are forced closer together by the spectral-reordering algorithm. The spectrum colour bar shown below the matrix represents the correspondence between the position of a voxel on this matrix and the projection of this position on to the brain to the right of the matrix. The brain images display the projection of each voxels position in the matrix on to the cortex. The structural connectivity patterns of the red voxels are most distinct from those of the purple voxels.


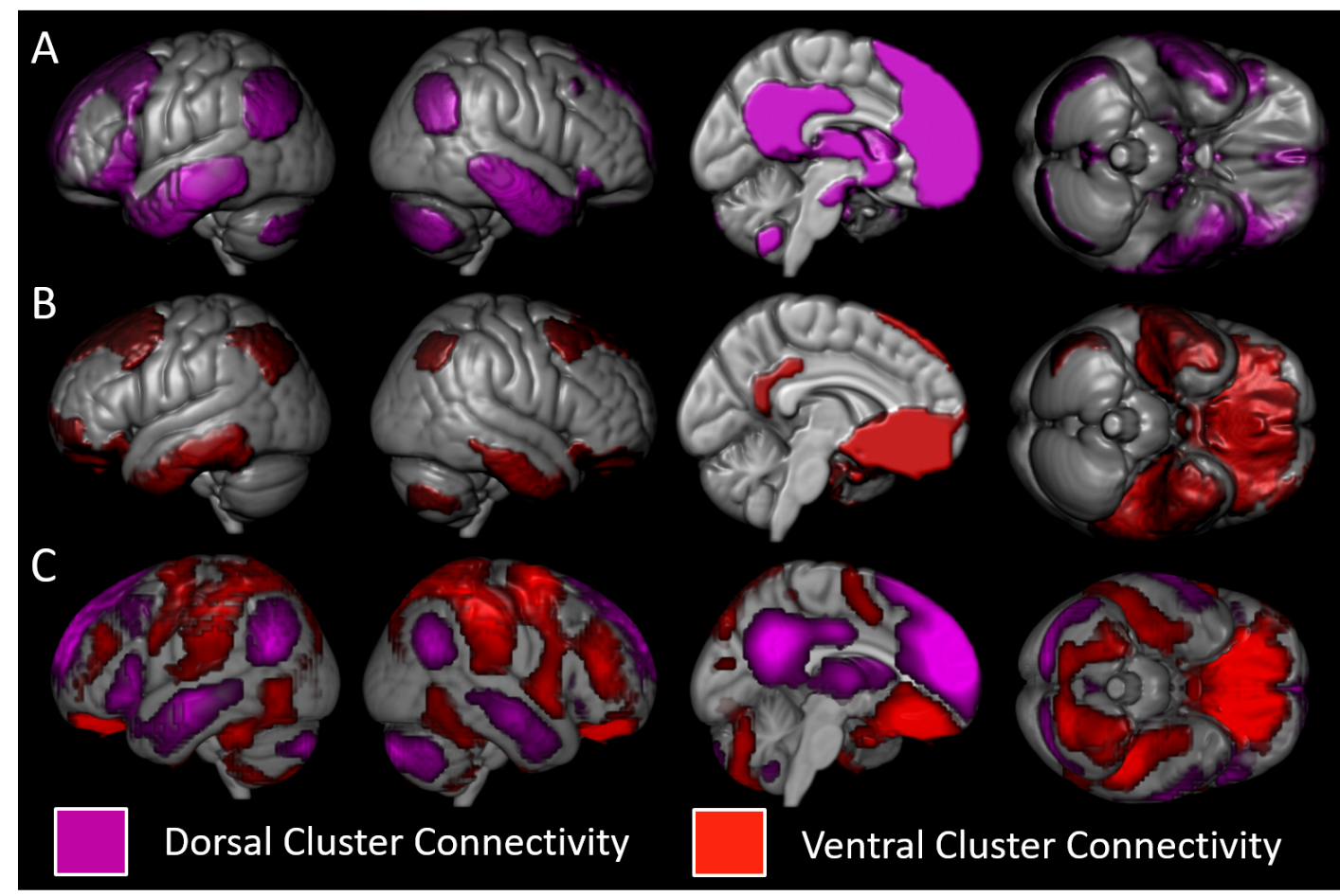


*Supplementary Figure 5.* Understanding the differential functional connectivity of the dorsal and ventral vmPFC in the context of their strong functional connections. A. The significant functional connectivity of the dorsal vmPFC cluster. B. The significant functional connectivity of the ventral vmPFC cluster. C. The differential functional connectivity of the dorsal (violet) and ventral (red) vmPFC is displayed. Unlike within the main manuscript, all differences are shown without masking by the significant functional connectivity of each region.

Supplementary Table 2. Differential functional connectivity of the two core distinct vmPFC clusters identified in the functional resting-state data.

| Contrast | Region of Activation | Cluster extent (voxels) | Max z value | P value (FWE corrected) | Peak MNI Coordinate | | |
| --- | --- | --- | --- | --- | --- | --- | --- |
|  |  |  |  |  | X | Y | Z |
| Purple (Dorsal) Cluster > Red (Ventral) Cluster | Bilateral dmPFC, ACC, IFG, insula, precuneus, PCC, thalamus & L MFG, pMTL, sTP, MTG & aITG | 10236 | Inf | >.001 | -3 | 57 | 15 |
|  | L AG | 758 | Inf | >.001 | -51 | -60 | 30 |
|  | Cerebellum | 679 | Inf | >.001 | 27 | -81 | -36 |
|  | Cerebellum | 195 | Inf | .002 | -30 | -84 | -36 |
|  | R AG | 423 | Inf | >.001 | 54 | -57 | 30 |
|  | R MTG, Heschl's gyrus & aITG | 773 | 6.77 | >.001 | 63 | -12 | -15 |
| Red (Ventral) Cluster > Purple (Dorsal) Cluster | Bilateral OFC, vATL, aPHG, pITC, cerebellum, occipital cortex, precuneus, SPL, SMG, SMA, motor cortex, insula, R STG | 16829 | Inf | >.001 | -12 | 36 | -21 |
|  | L MFG | 103 | 6.29 | .029 | -48 | 39 | 27 |

*Clusters significant at .05 after FWE correction. L = left, R = right, a = anterior, p = posterior, dmPFC = dorsomedial prefrontal cortex, IFG = inferior frontal gyrus, MFG = middle frontal gyrus, PCC = posterior cingulate cortex, MTL = medial temporal lobe, sTP = superior temporal pole, MTG = middle temporal gyrus, ITG = inferior temporal gyrus, AG = angular gyrus, OFC = orbitofrontal cortex, vATL = ventral anterior temporal lobe, PHG = parahippocampal gyrus, ITC = inferior temporal cortex, SPL = superior parietal lobe, SMG = supramarginal gyrus and SMA = supplementary motor area.*

Supplementary Table 3. Functional connectivity of each left vmPFC clusters identified in the hard parcellation of the functional resting-state data.

| Contrast | Region of Activation | Cluster extent (voxels) | Max z value | P value (FWE corrected) | Peak MNI Coordinate | | |
| --- | --- | --- | --- | --- | --- | --- | --- |
|  |  |  |  |  | X | Y | Z |
| Purple (Dorsal) Cluster | Bilateral dmPFC, ACC, IFG, insula, precuneus, PCC, thalamus, MTL, sTP, MTG & aITG | 13802 | Inf | >.001 | -18 | 60 | 18 |
|  | L AG | 835 | Inf | >.001 | -51 | -63 | 33 |
|  | Cerebellum | 823 | Inf | >.001 | 30 | -81 | -36 |
|  | R AG | 438 | Inf | >.001 | 54 | -60 | 33 |
|  | Cerebellum | 272 | Inf | >.001 | -30 | -84 | -36 |
|  | Cerebellum | 192 | Inf | .002 | 6 | -57 | -48 |
| Red (Ventral) Cluster | Bilateral OFC, vATL & aPHG | 6329 | Inf | >.001 | -12 | 36 | -18 |
|  | Bilateral MFG | 1004 | Inf | >.001 | -33 | 18 | 60 |
|  | L AG | 576 | 6.52 | >.001 | -42 | -69 | 42 |
|  | R AG | 137 | 5.09 | >.05 | 54 | -66 | 42 |
|  | Cerebellum | 218 | 5.05 | .001 | 45 | -66 | -42 |
|  | PCC | 131 | 4.44 | .005 | -6 | -54 | 18 |

*Clusters significant at .05 after FWE correction. L = left, R = right, a = anterior, dmPFC = dorsomedial prefrontal cortex, IFG = inferior frontal gyrus, MFG = middle frontal gyrus, ACC = anterior cingulate cortex, PCC = posterior cingulate cortex, MTL = medial temporal lobe, sTP = superior temporal pole, MTG = middle temporal gyrus, ITG = inferior temporal gyrus, AG = angular gyrus, OFC = orbitofrontal cortex, vATL = ventral anterior temporal lobe, PHG = parahippocampal gyrus.*
